# Associations of physical activity intensity, frequency, duration, and volume with the incidence of sarcopenia in middle-aged and older adults: a 4-year longitudinal study in China

**DOI:** 10.1186/s12877-024-04873-x

**Published:** 2024-03-16

**Authors:** Xiaoguang Zhao, Dongxue Liu, Hongjun Zhang, Shaoshuai Shen, Naipeng Zhang, Yihan Pan, Chao Fu, Wenjiao Wang, Hang Ren, Xiaopeng Pan

**Affiliations:** 1 Research Academy of Grand Health, Ningbo University, Ningbo, Zhejiang 315211 China; 2 Faculty of Sports Science, Ningbo University, Ningbo, Zhejiang 315211 China; 3 School of Physical Education, Liaoning Finance and Trade College, Xingcheng, Liaoning 125100 China; 4 School of Education and Welfare, Aichi Prefectural University, Nagakute, Aichi 480-1198 Japan

**Keywords:** Muscle mass, Muscle strength, Physical activity, Physical performance, Sarcopenia

## Abstract

**Background:**

Physical activity (PA) plays an important role in the process of several chronic diseases. It may be also associated with the incidence of sarcopenia. This study aimed to determine the association of PA from different components including frequency, duration, intensity, and volume with the incidence of sarcopenia in middle-aged and older adults.

**Methods:**

This study used data from the China Health and Retirement Longitudinal Study in 2011 and 2015. A total of 3,760 individuals aged ≥ 40 years were involved in this study. Sarcopenia was diagnosed using muscle mass, strength and physical performance according to the Asian Working Group for Sarcopenia. PA information including frequency, duration, intensity, and volume was obtained by a self-reported questionnaire. Logistic regression analysis was employed to examine the association between PA and the incidence of sarcopenia at 4-year follow-up.

**Results:**

The incidence of sarcopenia was 5.9% during the 4-year follow-up. Compared to sedentary individuals, those taking 1–2 days or more per week, or a minimum of 10 min each time on vigorous-intensity PA (VPA) had a lower incidence of sarcopenia. Adults spending 3 days or more each week, a minimum of 30 min each time, or 150 min or more per week on moderate-intensity PA (MPA) had a lower presence of sarcopenia than sedentary adults. Adults taking 3 days or more per week, at least 30 min each time, or 150 min or more each week on light-intensity PA (LPA) tended to have a lower incidence of sarcopenia than sedentary individuals. Sensitivity analyses confirmed the robustness of the findings after removing persons with hypertension, dyslipidemia, or diabetes.

**Conclusions:**

These findings suggest that the frequency, duration, and volume of VPA or MPA are negatively associated with the presence of sarcopenia. Participation in LPA tends to have a lower incidence of sarcopenia in middle-aged and older adults.

**Supplementary Information:**

The online version contains supplementary material available at 10.1186/s12877-024-04873-x.

## Introduction

Sarcopenia is a growing public health issue around the world. Sarcopenia is a common geriatric condition that is defined by an age-related loss of skeletal muscle mass, a drop in muscle strength and physical performance [1]. A previous study found that around the age of 40, muscle mass begins to diminish at a rate of 8% every decade [2]. Muscle strength and function are generally decreased alongside reductions in muscle mass. However, the decrease of muscle strength is far faster than the reduction of muscle mass [3]. Through a number of working group definitions, including the most recent one from the Asian Working Group on Sarcopenia in 2019 (AWGS 2019) [4], the definition of sarcopenia, which was formerly based on muscle mass loss, has been modified to highlight the deterioration in muscle strength and physical performance. A growing body of studies indicated that sarcopenia is linked to a range of adverse health outcomes, including frailty, functional disability, fractures, falls, morbidity, and mortality [5–7]. Sarcopenia poses a substantial impediment to healthy aging. As a result, it is critical to identify treatment targets for sarcopenia in order to avoid or delay its onset.

Inadequate physical activity (PA) is viewed as a primary contributor to sarcopenia. In a study of 162 older people between the ages of 60 and 80, Gianoudis et al. [8] discovered that spending more sedentary time is linked to a higher likelihood of sarcopenia, with the likelihood rising by 33% for every additional hour of sedentary behavior. Westbury and colleagues [9] indicated that older adults who engage in moderate-to-vigorous-intensity PA (MVPA) have a faster walking speed, a lower level of obesity, and a reduced chance of sarcopenia than those who engage in low-intensity PA (LPA). Physical exercise, especially resistance exercise can increase muscle mass and strength, as well as cardiovascular function and physical performance according to a range of studies [10–12], which can prevent or delay the development of sarcopenia in middle-aged and older adults.

PA is made up of four major components: frequency, duration, intensity, and weekly volume. There are specific guidelines on PA from a number of organizations. For example, the World Health Organization [13] recommends that older persons engage in 75 min or more per week of vigorous-intensity PA (VPA), 150 min or more per week of MPA, or an equivalent combination of MVP and VPA; the American College of Sports Medicine [14] further recommends that healthy individuals engage in at least 20 min per week of VPA each day on three different days, or at least 30 min per week of MPA on five different days. To the best of our knowledge, despite multiple publications on the link between PA, sarcopenia, and possible sarcopenia in middle-aged and older adults [15–17], the majority of previous studies used a cross-sectional design. Therefore, a longitudinal investigation is needed to determine the relationship between PA and sarcopenia. Moreover, to date, no research has investigated the association by taking into account all the four PA components such as frequency, duration, intensity, and weekly volume.

Thus, the aim of this prospective study was to explore the association of PA from different components including frequency, duration, intensity, and volume with the incidence of sarcopenia in middle-aged and older adults, using data from a national representative population-based sample in China.

## Materials and methods

### Study setting and participants

The data for this study came from the first and third waves of the China Health and Retirement Longitudinal Study (CHARLS), which were conducted in 2011 and 2015. The CHARLS survey aims to collect high-quality information on the demographic characteristics of non-institutionalized Chinese people in their middle and older years, as well as details on their families, health and functioning, lifestyle behaviors, health care, and employment and retirement. Detailed information regarding the CHARLS, as well as a description of its methodology, can be obtained on the official website (http://charls.pku.edu.cn/) and in published papers [18, 19].

**Fig. 1:**
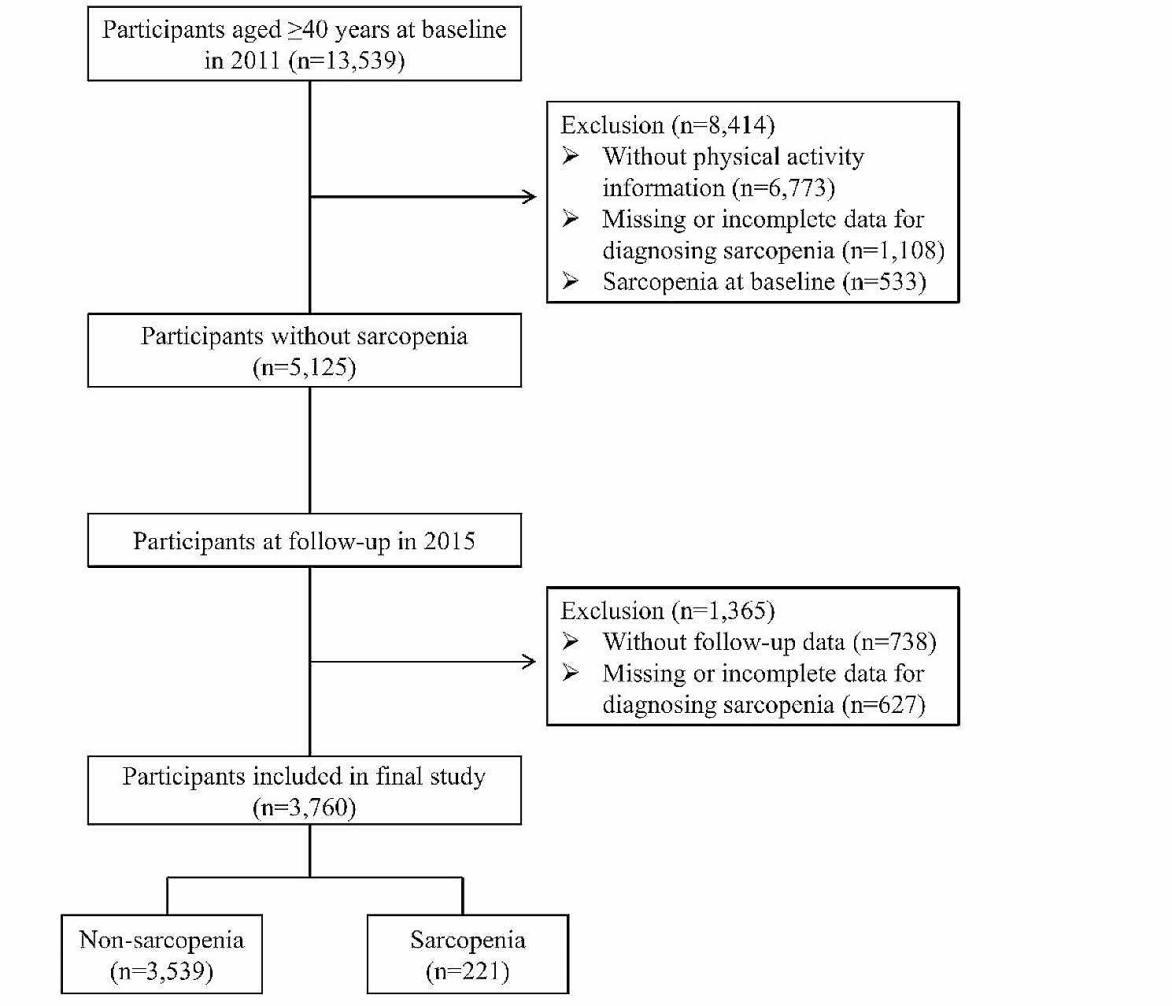
Flowchart of study participants in this study

In the first wave of the survey in 2011, a total of 13,539 community-dwelling adults aged ≥ 40 years were enrolled. We removed 8,414 people for the following reasons: (a) without PA information (*n* = 6,773); (b) incomplete or missing data for defining sarcopenia (*n* = 1,108); (c) having sarcopenia at baseline (*n* = 533). There were in total 5,125 participants who did not have sarcopenia in the final study population. The third wave was conducted in 2015, nearly 4 years after the first wave. The exclusion criteria were as follows: (a) without follow-up data (*n* = 738); and (b) incomplete or missing data for defining sarcopenia (*n* = 627). Consequently, a total of 3,760 middle-aged and older people aged from 40 to 94 years were included in the final analysis (Fig. 1). To assess the potential selection bias caused by missing data, variables such as age, gender, education levels, smoking status, and hypertension were chosen to compare the observed data group and the missing data group (Additional file 1).

### Assessment of PA

A modified version of the International Physical Activity Questionnaire (IPAQ) Short Form (Additional file 2) was used to measure PA. The modified questionnaire was structured and described similarly to the IPAQ. Previous studies have confirmed the validity and reliability of the IPAQ [20–22]. The procedure for evaluating the PA in our study was as follows: First, individuals were questioned about whether they had engaged in the intensity of PA for at least 10 min during a usual week continuously. The intensity of PA was specified as follows: (a) VPA: activities such as heavy lifting, digging, plowing, fast bicycling, cycling with a heavy load, and aerobics are examples of activities that cause a person to breathe much harder than usual; (b) MPA: activities such as carrying light loads, bicycling at a regular pace, and mopping the floor are examples of activities that cause a person to breathe somewhat harder than usual; (c) LPA: activities include walking at home or work, walking to get from one place to another, and any other walking done exclusively for sport, exercise, recreation or leisure. If the answer was “yes”, the participants were next asked how often (days/week, d/w) and how long (minutes/day, min/d) they engaged in the intensity of PA. The frequency of PA ranged from 0 to 7 d/w and was divided into three categories: sedentary, 1–2 d/w, and ≥ 3 d/w. The duration of PA was also divided into three scales: sedentary, 10–29 min/d, and ≥ 30–119 min/d. The weekly volume (minutes/week, min/w) of VPA/MPA/LPA was calculated using the following equation: Volume of VPA/MPA/LPA = Frequency of VPA/MPA/LPA × Duration of VPA/MPA/LPA. The weekly volume of VPA/MPA/LPA was divided into three groups: sedentary, < 150 min/w, and ≥ 150 min/w.

### Diagnosis of sarcopenia

Sarcopenia was identified using a combination of the loss of muscle mass and the deterioration in muscle strength or physical performance based on the AWGS 2019 criteria [4]. Sarcopenia was assessed in our study by measuring appendicular skeletal muscle mass (ASMM), muscle strength, and physical function. To calculate the ASMM, we employed an anthropometric equation for the Chinese population that had previously been validated. A previous study has found strong agreement between the dual X-ray absorptiometry and the anthropometric equation [23]. ASMM can be calculated from the following anthropometric equation: ASMM = 0.193 × body weight (kg) + 0.107 × body height (cm) − 4.157 × gender (male = 1, female = 2) − 0.037 × age (years) − 2.631. We adjusted the ASMM by dividing it by the square of height (ASMM/Height^2^), as in earlier research [24]. We utilized the sex-specific lowest 20% of the ASMM/Height^2^ as the cut-off to determine the low muscle mass, which was 8.02 kg/m^2^ for males and 6.46 kg/m^2^ for females. Measures of muscle strength and physical performance included the 5-time chair stand test and handgrip strength, respectively. According to the AWGS 2019, we classified low physical performance as a time of ≥ 12 s for completing the 5-time chair stand test, and low muscle mass as handgrip strength of < 28 kg for males and < 18 kg for females. The measurement protocols for the 5-time chair stand test and handgrip strength can be found in our previous publications [18, 25].

### Potential covariates

Our study’s covariates, which were chosen based on published papers [25–27], included sociodemographic characteristics, lifestyle-related factors, and health-related variables. The sociodemographic characteristics included age (40–49, 50–59, 60–69, and ≥ 70 years), gender (male and female), education levels (illiteracy, ≤primary school, middle school, and ≥ high school), and marital status (married and living with spouse, widowed, and others). Lifestyle-related factors encompassed alcohol drinking frequency (> 1/month, ≤1/month, and never drank) and smoking status (current smokers, former smokers, and never smoked). Health-related variables covered major chronic diseases (e.g., hypertension, dyslipidemia, and diabetes).

### Statistical analysis

The study participants’ characteristics were reported as numbers with percentages. To compare these characteristics between the non-sarcopenia and sarcopenia groups, we performed Chi-square tests. The incidence of sarcopenia at the follow-up years was investigated using logistic regression analysis, both with and without adjusting for age, gender, education levels, marital status, alcohol consumption frequency, smoking status, hypertension, dyslipidemia, and diabetes. In the logistic regression analysis, each VPA, MPA, and LPA used “sedentary” (no activity) as the reference group. The odds ratios (OR) and 95% confidence intervals (CI) were computed. We ran sensitivity analyses for major chronic disease-free participants, excluding people with hypertension, dyslipidemia, or diabetes, to confirm the robustness of our findings. In our study, Chi-square tests and logistic regression analyses were performed using SPSS Statistics version 26.0 (IBM Corp., Chicago, IL, USA).

## Results

The final analysis included 3,760 participants, and 221 of them had sarcopenia, with an incidence of 5.9% at 4-year follow-up. Table 1 depicts the differences in sociodemographic characteristics, lifestyle-related factors, and health-related variables between the non-sarcopenia and sarcopenia groups at the first wave. Compared to those without sarcopenia at baseline, those with new-onset sarcopenia are more likely to be older (28.9% vs. 42.1% for 60–69 years, 9.0% vs. 34.4% for ≥ 70 years, *p* < 0.001), widowed (8.1% vs. 17.2%, *p* < 0.001), and illiterate (26.2% vs. 41.2%, *p* < 0.001). However, we did not find significant differences in smoking status, alcohol consumption frequency, and major chronic diseases between the non-sarcopenia and sarcopenia groups.

**Table 1:** Baseline characteristics of participants according to the presence of sarcopenia

Variables	Non-sarcopenia (*n* = 3,539)	Sarcopenia (*n* = 221)	p value
Gender (*n* = 3,760)			0.404
Male	1,575 (44.5)	92 (41.6)	
Female	1,964 (55.5)	129 (58.4)	
Age (*n* = 3,760)			< 0.001
40–49 years	815 (23.0)	8 (3.6)	
50–59 years	1,381 (39.0)	44 (19.9)	
60–69 years	1,023 (28.9)	93 (42.1)	
≥70 years	320 (9.0)	76 (34.4)	
Marital status (*n* = 3,760)			< 0.001
Married and living with spouse	3,043 (86.0)	171 (77.4)	
Widowed	287 (8.1)	38 (17.2)	
Others	209 (5.9)	12 (5.4)	
Education levels (*n* = 3,760)			< 0.001
Illiterate	926 (26.2)	91 (41.2)	
≤primary school	688 (19.4)	46 (20.8)	
Elementary school	815 (23.0)	45 (20.4)	
Middle school	770 (21.8)	28 (12.7)	
≥high school	340 (9.6)	11 (5.0)	
Smoking status (*n* = 3,760)			0.764
Current smokers	1,011 (28.6)	68 (30.8)	
Former smokers	267 (7.5)	17 (7.7)	
Never smoked	2,261 (63.9)	136 (61.5)	
Alcohol drinking frequency (*n* = 3,760)			0.141
> 1/month	884 (25.0)	43 (19.5)	
≤1/month	270 (7.6)	21 (9.5)	
Never drank	2,385 (67.4)	157 (71.0)	
Hypertension (*n* = 3,740)			0.267
Yes	848 (24.1)	46 (20.8)	
No	2,671 (75.9)	175 (79.2)	
Dyslipidemia (*n* = 3,681)			0.322
Yes	341 (9.8)	17 (7.8)	
No	3,122 (90.2)	201 (92.2)	
Diabetes (*n* = 3,728)			0.764
Yes	192 (5.5)	11 (5.0)	
No	3,316 (94.5)	209 (95.0)	

Regarding PA frequency, it was found that taking 1–2 d/w or more (OR: 0.30, 95% CI: 0.10–0.88 for 1–2 d/w; OR: 0.45, 95% CI: 0.27–0.73 for ≥ 3 d/w) on VPA, or taking ≥ 3 d/w (OR: 0.59, 95% CI: 0.38–0.92) on MPA was negatively associated with the incidence of sarcopenia, even after adjustment with covariates. Spending ≥ 3 d/w (OR: 0.66, 95% CI: 0.43–1.01) on LPA tended to have a lower incidence of sarcopenia (Table 2). In terms of PA duration, we noticed that taking 10–29 min/d or more (OR: 0.39, 95% CI: 0.16–0.95 for 10–29 min/d; OR: 0.46, 95% CI: 0.28–0.74 for ≥ 30 min/d) on VPA, or spending ≥ 30 min/d (OR: 0.60, 95% CI: 0.38–0.93) on MPA was negatively associated with the progression of sarcopenia after adjustments. Taking ≥ 30 min/d (OR: 0.67, 95% CI: 0.43–1.03) on LPA tended to have a lower presence of sarcopenia (Table 3). When it comes to PA volume, we observed that taking < 150 min/w (OR: 0.48, 95% CI: 0.25–0.94) or ≥ 150 min/w (OR: 0.42, 95% CI: 0.25–0.69) on VPA, or taking ≥ 150 min/w (OR: 0.59, 95% CI: 0.38–0.92) on MPA was negatively associated with the presence of sarcopenia after adjustments. Spending ≥ 150 min/w (OR: 0.62, 95% CI: 0.43–1.02) on LPA tended to have a lower incidence of sarcopenia (Table 4).

**Table 2:** Association between PA frequency and the incidence of sarcopenia in middle-aged and older adults

PA frequency	Event/participants	Sarcopenia	
		OR (95% CI)	Adjusted OR^†^ (95% CI)
VPA			
Sedentary	27/314	1.00 (ref.)	1.00 (ref.)
1–2 d/w	7/144	0.38 (0.11–0.96)^*^	0.30 (0.10–0.88)^*^
≥3 d/w	56/1,325	0.47 (0.29–0.76)^**^	0.45 (0.27–0.73)^**^
MPA			
Sedentary	27/314	1.00 (ref.)	1.00 (ref.)
1–2 d/w	5/145	0.54 (0.23–1.28)	0.57 (0.24–1.36)
≥3 d/w	117/2,165	0.61 (0.39–0.94)^*^	0.59 (0.38–0.92)^*^
LPA			
Sedentary	27/314	1.00 (ref.)	1.00 (ref.)
1–2 d/w	4/97	0.28 (0.10–1.22)	0.29 (0.10–1.24)
≥3 d/w	174/2,973	0.67 (0.44–1.02)	0.66 (0.43–1.01)

*Note* OR, odds ratio; CI, confidential intervals; d/w, days/week; VPA, vigorous physical activity; MPA, moderate physical activity; LPA, light physical activity

^*^p < 0.05, ^**^p < 0.01

^†^Adjusted for gender, age, marital status, education levels, smoking status, alcohol drinking frequency, hypertension, dyslipidemia, and diabetes

**Table 3:** Association of PA duration with the presence of sarcopenia in middle-aged and older adults

PA duration	Event/participants	Sarcopenia	
		OR (95% CI)	Adjusted OR^†^ (95% CI)
VPA			
Sedentary	27/314	1.00 (ref.)	1.00 (ref.)
10–29 min/d	3/64	0.52 (0.15–0.97)^*^	0.39 (0.16–0.95)^*^
≥30 min/d	60/1,405	0.47 (0.30–0.76)^**^	0.46 (0.28–0.74)^**^
MPA			
Sedentary	27/314	1.00 (ref.)	1.00 (ref.)
10–29 min/d	9/171	0.59 (0.27–1.29)	0.51 (0.15–1.77)
≥30 min/d	114/2,130	0.61 (0.39–0.93)^*^	0.60 (0.38–0.93)^*^
LPA			
Sedentary	27/314	1.00 (ref.)	1.00 (ref.)
10–29 min/d	24/486	0.67 (0.44–1.03)	0.83 (0.55–1.26)
≥30 min/d	154/2,583	0.55 (0.31–0.98)^*^	0.67 (0.43–1.03)

*Note* OR, odds ratio; CI, confidential intervals; min/d, minutes/day; VPA, vigorous physical activity; MPA, moderate physical activity; LPA, light physical activity

^*^p < 0.05, ^**^p < 0.01

^†^ Adjusted for gender, age, marital status, education levels, smoking status, alcohol drinking frequency, hypertension, dyslipidemia, and diabetes

**Table 4:** Association between PA volume and the incidence of sarcopenia in middle-aged and older adults

PA volume	Event/participants	Sarcopenia	
		OR (95% CI)	Adjusted OR^†^ (95% CI)
VPA			
Sedentary	27/314	1.00 (ref.)	1.00 (ref.)
10–149 min/w	12/162	0.59 (0.38–0.90)^*^	0.48 (0.25–0.94)^*^
≥150 min/w	51/1,293	0.44 (0.27–0.71)^**^	0.42 (0.25–0.69)^**^
MPA			
Sedentary	27/314	1.00 (ref.)	1.00 (ref.)
10–149 min/w	17/325	0.85 (0.42–1.73)	0.85 (0.41–1.75)
≥150 min/w	105/1,969	0.60 (0.39–0.93)^*^	0.59 (0.38–0.92)^*^
LPA			
Sedentary	27/314	1.00 (ref.)	1.00 (ref.)
10–149 min/w	31/576	0.61 (0.35–1.03)	0.60 (0.35–1.04)
≥150 min/w	147/2,487	0.67 (0.44–1.03)	0.62 (0.43–1.02)

*Note* OR, odds ratio; CI, confidential intervals; min/w, minutes/week; VPA, vigorous physical activity; MPA, moderate physical activity; LPA, light physical activity

^*^p < 0.05, ^**^p < 0.01

^†^Adjusted for gender, age, marital status, education levels, smoking status, alcohol drinking frequency, hypertension, dyslipidemia, and diabetes

We conducted sensitivity analyses for participants without major chronic diseases by excluding those who had diabetes, dyslipidemia, or hypertension (Additional file 3). The findings of the sensitivity analyses, which reveal an association between PA volume and the progression of sarcopenia, were found to be similar to the findings in Table 4.

## Discussion

To our knowledge, this is the first longitudinal study to determine the association of PA from different components including intensity, frequency, duration, and weekly volume with the progression of sarcopenia. Results indicated that the incidence of sarcopenia was 5.9% during the period between baseline and 4-year follow-up. Compared to sedentary individuals, those taking 1–2 days or more per week, or a minimum of 10 min each time on VPA had a lower incidence of sarcopenia. Adults spending 3 days or more each week, a minimum of 30 min each time, or 150 min or more per week on MPA had a lower presence of sarcopenia than sedentary adults. Adults taking 3 days or more per week, at least 30 min each time, or 150 min or more each week on LPA were inclined to have a lower incidence of sarcopenia than sedentary individuals.

Sarcopenia was shown to have a 5.9% incidence between baseline and the 4-year follow-up, according to the current study. The finding is comparable with earlier prospective studies, which found that sarcopenia incidence was 1.14% annually among the older population in English [28] and 1.06% annually among older persons living in communities in Japan [29]. In a population of 65 years of age and older, Yu and colleagues [30] observed a 4-year incidence of the proportion of 7.8% sarcopenia. Additionally, we did not find significant differences in the incidence of sarcopenia between genders in our study, which is consistent with a previous study that found no statistical differences in the prevalence of sarcopenia between males and females in the Vietnamese population [31].

Previous research has shown an association between MVPA and sarcopenia or its components. Marini et al. [32] performed a cross-sectional study to examine the role of PA level and sitting time as predictors of sarcopenia odds, discovering that time spent in MPA can prevent sarcopenia in community-dwelling older women. By using accelerometers to measure PA to explore its association with sarcopenia, Scott and colleagues [33] discovered that regardless of the length of amounts or bouts of sedentary behavior, a greater level of MVPA is consistently associated with a lower risk of sarcopenia and its components. A longitudinal study conducted by Mijnarends et al. [34] showed that although MVPA does not affect the rate of decline in muscle mass and function, it can delay the presence of sarcopenia in adults aged 66–93 years. Our findings support these previous results but additionally show what frequency, duration, and weekly volume of MVPA may decrease the incidence of sarcopenia in middle-aged and older adults. Specifically, spending 3 days or more each week, a minimum of 30 min each time, or 150 min or more each week on MPA, or taking 1–2 days or more each week, a minimum of 10 min each time, or 10–149 min or more each week on VPA associated with a lower incidence of sarcopenia. Compared to only providing information about PA intensity like previous studies, our study’s specific recommendation including PA intensity, frequency, duration, and weekly volume may be more practicable in preventing or treating sarcopenia.

It has been reported that not only MVPA, but LPA is also associated with a lower rate of sarcopenia or decreased sarcopenia risk such as low muscle mass, strength, and physical performance [25, 35–37]. However, there have also been previous studies suggesting that no significant relationship exists between LPA and sarcopenia in middle-aged and older individuals [33, 38, 39]. In the present study, although no statistical significance was detected, we observed that individuals taking 3 days or more per week, a minimum of 30 min each time, or 150 min or more per week on LPA were inclined to have a lower incidence of sarcopenia than sedentary adults. Additionally, it should be emphasized that LPA has been demonstrated to affect a range of health outcomes, including cognitive health, cardiovascular diseases, and mortality [40, 41]. Based on the above results, we believe that it may be helpful to increase LPA to prevent and treat sarcopenia and other chronic conditions, especially for older adults who have difficulty participating in MVPA.

PA plays an important role in the process of various chronic diseases. The findings of the present study suggest that increasing VPA or MPA may benefit in the prevention of sarcopenia. Resistance training may be useful in achieving this goal since it can successfully improve muscle mass, strength, and physical function, thereby decreasing the risk of sarcopenia [42–44]. In light of these, practitioners should be aware of the relationship between MVPA and sarcopenia and consider formulating resistance training strategies for sedentary people or those who only participate in LPA to aid in sarcopenia prevention. Future research should examine the PA-sarcopenia mechanism, as well as whether increasing MVPA for sedentary people or those who only take part in LPA can lead to a lower risk of sarcopenia onset.

The current study had some drawbacks. First, a modified version of IPAQ was used to gather data on PA rather than objective tools. A previous study has shown that when compared to objective PA measuring tools like accelerometers, the subjective IPAQ overrates levels of PA [45]. Moreover, the modified version of IPAQ defined LPA as activities such as walking at home or work, walking to get from one place to another, and any other walking done exclusively for sport, exercise, recreation or leisure. The definition might cause participants to misclassify brisk walking during sport and exercise that belongs to MVPA as LPA to some extent. Third, despite controlling for a range of relevant covariates, our study did not control for other potential factors such as dietary status and nutritional supplements, which could influence the relationship between PA and sarcopenia. Fourth, rather than using dual X-ray absorptiometry or bioelectrical impedance analysis to assess muscle mass, we used an anthropometric equation that had previously been validated in the Chinese population [23]. Fifth, the 4-year follow-up in our study was a relatively short period, which could have led to a minimal change in health status during the short observation period. Finally, more than half of the participants (8,414 of 13,539) were eliminated due to missing data at baseline, which might cause some selection bias. However, sensitivity analyses confirmed the findings’ robustness after excluding individuals with hypertension, dyslipidemia, or diabetes.

## Conclusions

In summary, the current study indicated that the incidence of sarcopenia was 5.9% during the period between baseline and 4-year follow-up. We found that individuals taking 1–2 days or more each week, a minimum of 10 min each time, or 10–149 min or more each week on VPA had a lower incidence of sarcopenia than sedentary persons; adults spending 3 days or more each week, a minimum of 30 min each time, or 150 min or more each week on MPA had a lower incidence of sarcopenia when compared to sedentary people; adults taking 3 days or more per week, at least 30 min each time, or 150 min or more per week on LPA were inclined to have a lower incidence of sarcopenia from baseline to 4-year follow-up.

These findings suggest that the frequency, duration, and volume of VPA or MPA are negatively associated with the presence of sarcopenia. Participation in LPA tends to have a lower incidence of sarcopenia in middle-aged and older adults.

### Electronic supplementary material

Below is the link to the electronic supplementary material.

**Supplementary Material 1:** The characteristics of study participants at baseline in 2011 according to the missing data

**Supplementary Material 2:** The modified IPAQ short form

**Supplementary Material 3:** Association between PA volume and the presence of sarcopenia in disease-free people

## Data Availability

The data that support the findings of this study are available in Peking University Open Research Data at http://charls.pku.edu.cn/en/index.htm.

## Abbreviations

ASMM: Appendicular Skeletal Muscle Mass
AWGS: Asian Working Group on Sarcopenia
CHARLS: China Health and Retirement Longitudinal Study
CI: Confidence Intervals
IPAQ: International Physical Activity Questionnaire
LPA: Light-intensity Physical Activity
MPA: Moderate-intensity Physical Activity
MVPA: Moderate-to-Vigorous-intensity Physical Activity
OR: Odds Ratios
PA: Physical Activity
VPA: Vigorous-intensity Physical Activity
