# Supplementary material for: Associations of physical activity intensity, frequency, duration, and volume with the incidence of sarcopenia in middle-aged and older adults: a 4-year longitudinal study in China
Source: BMC Geriatr. 2024 Mar 16;24:258. doi: 10.1186/s12877-024-04873-x (PMC10944603; doi:10.1186/s12877-024-04873-x)
Supplement: Supplementary file 1 — Supplementary Material 1: The characteristics of study participants at baseline in 2011 according to the missing data [file 12877_2024_4873_MOESM1_ESM.docx]

| **Additional file 1.** The characteristics of study participants at baseline in 2011 according to the missing data | | | |
| --- | --- | --- | --- |
|  | Observed data | Missing data | p value |
| Gender (n=13,539) |  |  | 0.620 |
| Male | 2,401 (46.8) | 3,905 (46.4) |  |
| Female | 2,724 (53.2) | 4,509 (53.6) |  |
| Age (n=13,539) |  |  | <0.001 |
| 40-49 years | 1,109 (21.6) | 1,869 (22.2) |  |
| 50-59 years | 1,822 (35.6) | 2,888 (34.3) |  |
| 60-69 years | 1,476 (28.8) | 2,173 (25.8) |  |
| ≥70 years | 718 (14.0) | 1,481 (17.6) |  |
| Marital status (n=13,539) |  |  | <0.001 |
| Married and living with spouse | 4,271 (83.3) | 6,344 (75.4) |  |
| Widowed | 529 (10.3) | 1,029 (12.2) |  |
| Others | 325 (6.3) | 1,041 (12.4) |  |
| Education levels (n=13,521) |  |  | <0.001 |
| Illiterate | 1,383 (27.0) | 2,495 (29.7) |  |
| ≤primary school | 980 (19.1) | 1,475 (17.6) |  |
| Elementary school | 1,151 (22.5) | 1,731 (20.6) |  |
| Middle school | 1,054 (20.6) | 1,681 (20.0) |  |
| ≥high school | 556 (10.9) | 1,015 (12.1) |  |
| Smoking status (n=13,525) |  |  | <0.001 |
| Current smokers | 1,536 (30.0) | 2,766 (32.9) |  |
| Former smokers | 435 (8.5) | 575 (6.8) |  |
| Never smoked | 3,154 (61.5) | 5,059 (60.2) |  |
| Alcohol drinking frequency (n=13,519) |  |  | 0.504 |
| >1/month | 1,303 (25.4) | 2,089 (24.9) |  |
| ≤1/month | 411 (8.0) | 641 (7.6) |  |
| Never drank | 3,411 (66.6) | 5,664 (67.5) |  |
| Hypertension (n=13,512) |  |  | <0.001 |
| Yes | 1.195 (23.4) | 861 (10.2) |  |
| No | 3.903 (76.6) | 7,553 (89.8) |  |
| Dyslipidemia (n=13,434) |  |  | <0.001 |
| Yes | 457 (9.1) | 0 (0.0) |  |
| No | 4,563 (90.9) | 8,414 (100.0) |  |
| Diabetes (n=13,466) |  |  | <0.001 |
| Yes | 274 (5.4) | 248 (3.0) |  |
| No | 4,803 (94.6) | 8,141 (97.0) |  |
