# Supplementary material for: Associations of physical activity intensity, frequency, duration, and volume with the incidence of sarcopenia in middle-aged and older adults: a 4-year longitudinal study in China
Source: BMC Geriatr. 2024 Mar 16;24:258. doi: 10.1186/s12877-024-04873-x (PMC10944603; doi:10.1186/s12877-024-04873-x)
Supplement: Supplementary file 2 — Supplementary Material 2: The modified IPAQ short form [file 12877_2024_4873_MOESM2_ESM.docx]

**Additional file 2.** The Modified IPAQ Short Form

| **PHYSICAL ACTIVITIES**  **(KKTYPE)** | | **DA051.** | **DA052.** |  | |
| --- | --- | --- | --- | --- | --- |
|  |  | During a usual week, did you do any [….] for at least 10 minutes continuously? | During a usual week, on how many days did you do [….] for at least 10 minutes? | How much time did you usually spend doing [….] on one of those days? | |
| **A.** | Now think about all the **vigorous activities** requiring hard/high-intensity physical effort that you do in **a usual week**. Vigorous activities make you breathe much harder than normal and may include heavy lifting, digging, plowing, aerobics, fast bicycling, and cycling with a heavy load. Think only about those physical activities that you did for at least 10 minutes at a time. | 3. No🡻  1. Yes🡺 | **└──┘**1..7 days | **DA053**  1. < 2 hours | **DA054**  1. < 30 minutes  2. ≥ 30 minutes |
|  |  |  |  | 2. ≥ 2 hours | **DA055**  3. < 4 hours  4. ≥ 4 hours |
| **B.** | Now think about activities which take **moderate physical effort** that you do in a usual week. Moderate physical activities make you breathe somewhat harder than normal and may include carrying light loads, bicycling at a regular pace, or mopping the floor. Again, think about only those physical activities that you did for at least 10 minutes at a time. | 3. No🡻  1. Yes🡺 | **└──┘**1..7 days | **DA053**  1. < 2 hours | **DA054**  1. < 30 minutes  2. ≥ 30 minutes |
|  |  |  |  | 2. ≥ 2 hours | **DA055**  3. < 4 hours  4. ≥ 4 hours |
| **C.** | Now think about the time you spend **walking** in a usual week. This includes at work and at home, walking to travel from place to place, and any other walking that you might do solely for recreation, sport, exercise, or leisure. | 3. No🡻  1. Yes🡺 | **└──┘**1..7 days | **DA053**  1. < 2 hours | **DA054**  1. < 30 minutes  2. ≥ 30 minutes |
|  |  |  |  | 2. ≥ 2 hours | **DA055**  3. < 4 hours  4. ≥ 4 hours |
