# Supplementary material for: Associations of physical activity intensity, frequency, duration, and volume with the incidence of sarcopenia in middle-aged and older adults: a 4-year longitudinal study in China
Source: BMC Geriatr. 2024 Mar 16;24:258. doi: 10.1186/s12877-024-04873-x (PMC10944603; doi:10.1186/s12877-024-04873-x)
Supplement: Supplementary file 3 — Supplementary Material 3: Association between PA volume and the presence of sarcopenia in disease-free people [file 12877_2024_4873_MOESM3_ESM.doc]

| **Additional file 3.** Association between PA volume and the presence of sarcopenia in disease-free people | | |
| --- | --- | --- |
| PA volume | Sarcopenia | |
|  | OR (95% CI) | Adjusted OR^†^ (95% CI) |
| VPA |  |  |
| Sedentary | 1.00 (ref.) | 1.00 (ref.) |
| 10-149 min/w | 0.50 (0.25 - 0.96)^*^ | 0.51 (0.26 - 0.98)^*^ |
| ≥150 min/w | 0.38 (0.23 - 0.64)^**^ | 0.40 (0.24 - 0.66)^**^ |
| MPA |  |  |
| Sedentary | 1.00 (ref.) | 1.00 (ref.) |
| 10-149 min/w | 0.80 (0.39 - 1.62) | 0.82 (0.40 - 1.68) |
| ≥150 min/w | 0.54 (0.34 - 0.84)^*^ | 0.56 (0.36 - 0.87)^*^ |
| LPA |  |  |
| Sedentary | 1.00 (ref.) | 1.00 (ref.) |
| 10-149 min/w | 0.60 (0.37 - 1.05) | 0.64 (0.39 - 1.08) |
| ≥150 min/w | 0.62 (0.40 - 0.95)^*^ | 0.65 (0.42 - 1.02) |

Note: OR, odds ratio; CI, confidential intervals; min/w, minutes/week; VPA, vigorous physical activity; MPA, moderate physical activity; LPA, light physical activity

^*^ p < 0.05, ^**^ p < 0.01

^†^ Adjusted for gender, age, marital status, education levels, smoking status, and alcohol drinking frequency
